# Association between a remimazolam–propofol combination for maintenance of anesthesia and extubation time: a propensity score analysis

**DOI:** 10.1186/s40981-025-00836-2

**Published:** 2025-12-04

**Authors:** Takayuki Katsuragawa, Soichiro Mimuro, Hiroki Anezaki, Yuji Suzuki, Tsunehisa Sato, Yoshitaka Aoki, Masakazu Yamaguchi, Yoshiki Nakajima

**Affiliations:** 1 Department of Anesthesiology, Iwata City Hospital, 512-3 Okubo, Iwata, Shizuoka, 438-8550 Japan; 2 Department of Anesthesiology and Intensive Care Medicine, Hamamatsu University School of Medicine, 1-20-1 Handayama, Chuo-Ku, Hamamatsu, Shizuoka, 431-3192 Japan

**Keywords:** Remimazolam, Propofol, Non-cardiac surgery, Extubation time, Propensity score matching

## Abstract

**Background:**

This study aimed to evaluate the effect of combined administration of remimazolam and propofol on extubation time during anesthesia maintenance.

**Methods:**

This retrospective study was conducted at Hamamatsu University Hospital, enrolling adult patients who underwent non-cardiac surgery between September 2020 and October 2024. Eligible patients underwent invasive arterial pressure monitoring and anesthesia maintenance with remimazolam alone (RB group), propofol alone (PROP group), or a combination of both (RB + PROP group). Extubation time was defined as the interval between the cessation of sedative administration and tracheal extubation. Propensity score matching was performed after adjusting for age, sex, body mass index, American Society of Anesthesiologists Physical Status, preoperative comorbidities, type of surgery, combined with epidural anesthesia, scheduled or emergency surgery, operation time, and blood loss. The primary endpoint was the extubation time, while the secondary endpoints included the severity of hypotension, assessed by the time-weighted average area under the threshold, and the incidence of postoperative nausea and vomiting.

**Results:**

The study included 165, 403, and 178 patients in the RB, PROP, and RB + PROP groups, respectively. After propensity score matching, 75 matched cohorts were analyzed. Among the three groups, significant differences were found in terms of extubation time and hypotension (*p* < 0.001 and *p* = 0.04, respectively), whereas the incidence of postoperative nausea and vomiting did not significantly differ (*p* = 0.23). In multiple comparisons, the RB group (9.0 min) and the RB + PROP group (9.0 min) had significantly shorter extubation times than the PROP group (13.0 min) (each *p* < 0.001). Time-weighted average area under the threshold was significantly lower in the RB group (0.74 mmHg) than in the PROP group (2.03 mmHg) (*p* = 0.03).

**Conclusion:**

Extubation time with combined remimazolam and propofol was comparable to that with remimazolam alone, and both were shorter than that with propofol alone.

**Supplementary Information:**

The online version contains supplementary material available at 10.1186/s40981-025-00836-2.

## Background

Early extubation following general anesthesia is an important determinant of perioperative outcomes [1, 2]. Even modest reductions in emergence and recovery times can decrease operating room occupancy and enhance perioperative efficiency [3]. Therefore, the selection of suitable anesthetic agents that facilitate rapid and reliable recovery has substantial clinical value in surgical practice.

Remimazolam is a benzodiazepine-type sedative with a faster onset than midazolam, producing inactive metabolites that result in rapid recovery and a predictable duration of action [4, 5]. The sedative effects of this drug can be antagonized by flumazenil [6]. Compared to propofol, remimazolam is associated with a lower incidence of hypotension [7, 8]. However, sedation with remimazolam alone may increase body movements [9]. Conversely, propofol is widely used due to its favorable pharmacokinetic properties [10], even though it can cause hemodynamic instability due to vasodilation and decreased myocardial contractility, and its administration has been associated with reduced survival in critically ill patients compared to other sedatives [11]. Previous studies have indicated that the co-administration of remimazolam and propofol can improve sedation quality and safety [9]. This combination may reduce drug requirements, improve cardiovascular stability, and lower the incidence of intraoperative hypotension [12, 13].

Although remimazolam is associated with delayed awakening compared to propofol, the use of flumazenil may facilitate faster emergence, which could be advantageous in clinical practice [14, 15]. A case series suggested that combining remimazolam with propofol during general anesthesia may allow for dose reduction of each agent and potentially shorten the recovery time [16].

However, the effect of combining remimazolam and propofol on awakening time during general anesthesia remains unclear. Therefore, in this study, we investigated the effect of remimazolam–propofol co-administration on the recovery time from anesthesia in patients undergoing non-cardiac surgery.

## Methods

### Study design

This single-center retrospective study was approved by the Ethics Committee of Hamamatsu University Hospital, Hamamatsu, Japan (approval number 24–272). Since this was a retrospective study, the requirement for informed consent was waived by the Ethics Committee.

### Patient selection

We included adult patients who underwent non-cardiac surgery at Hamamatsu University Hospital between September 2020 and October 2024, during which invasive arterial pressure monitoring was performed and anesthesia was maintained with remimazolam (RB group), propofol (PROP group), or a combination of both agents (RB + PROP group). Patients who received inhalational anesthetics during surgery were excluded.

The choice of the anesthetic agent, target depth of anesthesia, and hemodynamic management were determined by the attending anesthesiologist. The use of a target-controlled infusion (TCI) system for propofol administration was at their discretion, in which case either a Terufusion Syringe Pump TE-371 or a Terufusion Syringe Pump Type SS 3TCI TE-SS830T (Terumo Co., Tokyo, Japan) was employed. The Diprifusor TCI system (AstraZeneca Plc., London, UK) is built into these pumps and operates according to a multicompartment pharmacokinetic model, known as the Marsh model [17]. Electroencephalographic (EEG) monitoring was performed as needed using either SedLine® (Masimo Corporation, Irvine, CA, USA) or Bispectral Index™ (Medtronic Inc., Minneapolis, MN, USA). The target ranges for the Bispectral Index (BIS) and Patient State Index (PSI) were maintained at 40–60 and 25–50, respectively.

Patients were excluded if the surgery was canceled, extubation was not performed, sedation was continued at the time of extubation, the duration of invasive arterial pressure monitoring was ≤ 10 min, or if clinical data were incomplete.

### Perioperative variables

The following baseline characteristics were recorded: age, sex, body mass index (BMI), American Society of Anesthesiologists Physical Status (ASA-PS), and comorbidities (hypertension, coronary artery disease, cerebrovascular disease, and diabetes mellitus). Additional perioperative data included the type of surgery, combined with epidural anesthesia, whether the surgery was scheduled or emergency, operation time, blood loss, anesthesia time, intravenous fluid volume, and urine output. Anesthetic drug usage was assessed, including the mean infusion rates of remimazolam, propofol, remifentanil, and the total fentanyl dose. The use of flumazenil and the dosage of vasopressors (ephedrine, phenylephrine, dopamine, and norepinephrine) were also recorded.

### Outcome variables

The primary outcome was defined as the extubation time, measured from discontinuation of the anesthetic maintenance drug used during surgery to extubation. Extubation was performed according to the institutional criteria after confirming the resolution of muscle relaxation, the presence of adequate spontaneous respiration, and the patient’s ability to open their eyes and follow verbal commands upon emergence from anesthesia.

The secondary outcome measures included the incidence of intraoperative hypotension and postoperative nausea and vomiting (PONV), i.e., nausea and/or vomiting occurring within 24 h of general anesthesia [18].

Hypotension was defined as a mean arterial pressure (MAP) of < 65 mmHg. The area under the MAP curve below 65 mmHg (expressed in mmHg·min) was calculated and divided by the duration of surgery to obtain the time-weighted average area under the threshold (TWA-AUT) (mmHg) [19]. Invasive arterial pressure was measured at one-minute intervals, and artifacts were corrected based on previously published methods [20].

### Statistical analysis

Continuous variables are expressed as the mean (standard deviation) or median (interquartile range [IQR]), as appropriate, and categorical variables are expressed as counts (percentages). The normality of continuous variables was assessed using the Shapiro–Wilk test. For normally distributed data, differences among the three groups were tested using one-way analysis of variance. For non-normally distributed data, the Kruskal–Wallis test was used. Pairwise comparisons among the three groups were performed using the Steel–Dwass test. Categorical variables were compared using the chi-squared test or Fisher’s exact test, as appropriate.

Propensity score matching was used to minimize bias in estimating treatment effects by matching participants with similar distributions of confounding variables. The following potential confounders were used to calculate propensity scores: age, sex, BMI, ASA-PS, hypertension, coronary artery disease, cerebrovascular disease, diabetes mellitus, type of surgery, combined with epidural anesthesia, scheduled or emergency surgery, operation time, and blood loss.

Propensity score matching was performed using the optimal matching method at a 1:1:1 ratio. All statistical analyses were conducted using the R software (version 4.3.0; R Development Core Team, Vienna, Austria). The R package TriMatch was used for propensity score matching. Statistical significance was set at *P* < 0.05. For chi-square tests involving multiple comparisons of categorical data, the alpha level was adjusted to 0.017 using the Bonferroni correction.

## Results

### Patients

**Fig. 1:**
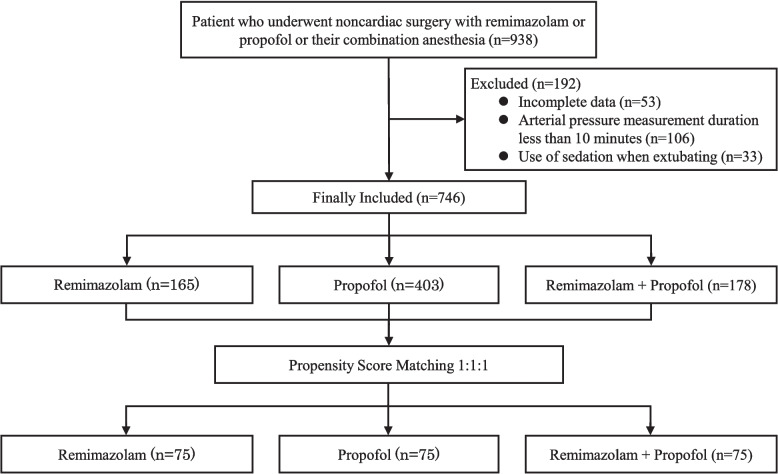
Flow diagram of the study

Between September 2020 and December 2024, a total of 938 patients were screened. Fifty-three cases were excluded due to the absence of BMI data, 106 cases because of a blood pressure measurement time of less than 10 min, and 33 cases due to the continued administration of sedatives until extubation. Thus, 746 patients were included in the final analysis. Propensity score matching yielded 75 patients in each group (Fig. 1). Before matching, significant differences were observed in age, sex, BMI, coronary artery disease, and the proportion of scheduled versus emergency surgeries. After matching, no significant differences were observed, and the groups were considered well-balanced (Table 1).

**Table 1:** Patient characteristics before and after propensity score matching with included variables as covariates

	Total cohort				Propensity score-matched cohort			
	Group RB	Group PROP	Group RB + PROP	P	Group RB	Group PROP	Group RB + PROP	P
	(*n* = 165)	(*n* = 403)	(*n* = 178)		(*n* = 75)	(*n* = 75)	(*n* = 75)	
Age (year)	74.0 (67.0–81.0)	65.0 (51.0–74.0)	69.5 (55.0–75.0)	< 0.001	72.0 (56.5–78.0)	73.0 (63.0–76.0)	71.0 (66.0–75.0)	0.94
Male	85 (51.5%)	157 (39.0%)	81 (45.5%)	0.02	31 (41.3%)	36 (48.0%)	33 (44.0%)	0.71
BMI (kg/m2)	21.6 (19.3–24.3)	22.9 (20.6–25.3)	22.7 (20.6–26.0)	0.001	22.8 (19.8–24.8)	22.7 (20.7–24.9)	22.0 (20.2–24.4)	0.76
ASA-PS				< 0.001				0.30
Class 1	8 (4.8%)	46 (11.4%)	12 (6.7%)		7 (9.3%)	6 (8.0%)	3 (4.0%)	
Class 2	93 (56.4%)	303 (75.2%)	141 (79.2%)		53 (70.7%)	56 (74.7%)	64 (85.3%)	
Class 3	63 (38.2%)	54 (13.4%)	25 (14.0%)		15 (20.0%)	13 (17.3%)	8 (10.7%)	
Class 4	1 (0.6%)	0 (0.0%)	0 (0.0%)		0 (0.0%)	0 (0.0%)	0 (0.0%)	
Comorbidities								
Hypertension	69 (41.8%)	140 (34.7%)	65 (36.5%)	0.28	29 (38.7%)	25 (33.3%)	26 (34.7%)	0.78
CAD	22 (13.3%)	15 (3.7%)	9 (5.1%)	< 0.001	4 (5.3%)	3 (4.0%)	4 (5.3%)	> 0.99
CVD	11 (6.7%)	27 (6.7%)	12 (6.7%)	> 0.99	2 (2.7%)	6 (8.0%)	1 (1.3%)	0.15
Diabetes mellitus	28 (17.0%)	46 (11.4%)	22 (12.4%)	0.19	7 (9.3%)	8 (10.7%)	9 (12.0%)	0.87
Operative profiles								
Type of surgery				< 0.001				0.21
General surgery	30 (18.2%)	19 (4.7%)	19 (10.7%)		11 (14.7%)	4 (5.3%)	8 (10.7%)	
Gynecology Surgery	8 (4.8%)	58 (14.4%)	12 (6.7%)		4 (5.3%)	7 (9.3%)	6 (8.0%)	
Hepatobiliary Surgery	8 (4.8%)	4 (1.0%)	2 (1.1%)		1 (1.3%)	1 (1.3%)	1 (1.3%)	
Neurosurgery	11 (6.7%)	75 (18.6%)	28 (15.7%)		6 (8.0%)	9 (12.0%)	12 (16.0%)	
Orthopedic surgery	49 (29.7%)	116 (28.8%)	64 (36.0%)		23 (30.7%)	25 (33.3%)	27 (36.0%)	
Other surgery	11 (6.7%)	17 (4.2%)	11 (6.2%)		1 (1.3%)	1 (1.3%)	5 (6.7%)	
Thoracic surgery	27 (16.4%)	78 (19.4%)	29 (16.3%)		17 (22.7%)	21 (28.0%)	9 (12.0%)	
Urological surgery	21 (12.7%)	36 (8.9%)	13 (7.3%)		12 (16.0%)	7 (9.3%)	7 (9.3%)	
Combined with epidural anesthesia	30 (18.2%)	61 (15.1%)	24 (13.5%)	0.47	11 (14.7%)	12 (16.0%)	14 (18.7%)	0.80
Scheduled surgery	152 (92.1%)	395 (98.0%)	175 (98.3%)	0.001	72 (96.0%)	72 (96.0%)	72 (96.0%)	> 0.99
Operation time (min)	149 (100–237)	185 (134–254)	186 (127–252)	0.02	149 (118–217)	166 (122–245)	188 (133–245)	0.26
Blood loss (mL)	58 (10–200)	40 (11–130)	35 (10–115)	0.16	41 (10–137)	25 (15–160)	30 (10–134)	0.99

Data are expressed as median (interquartile range), or number of patients (%)

*BMI* Body mass index, *ASA-PS* American Society of Anesthesiologists physical status, *CAD* Coronary artery disease, *CVD* Cerebrovascular disease

**Fig. 2:**
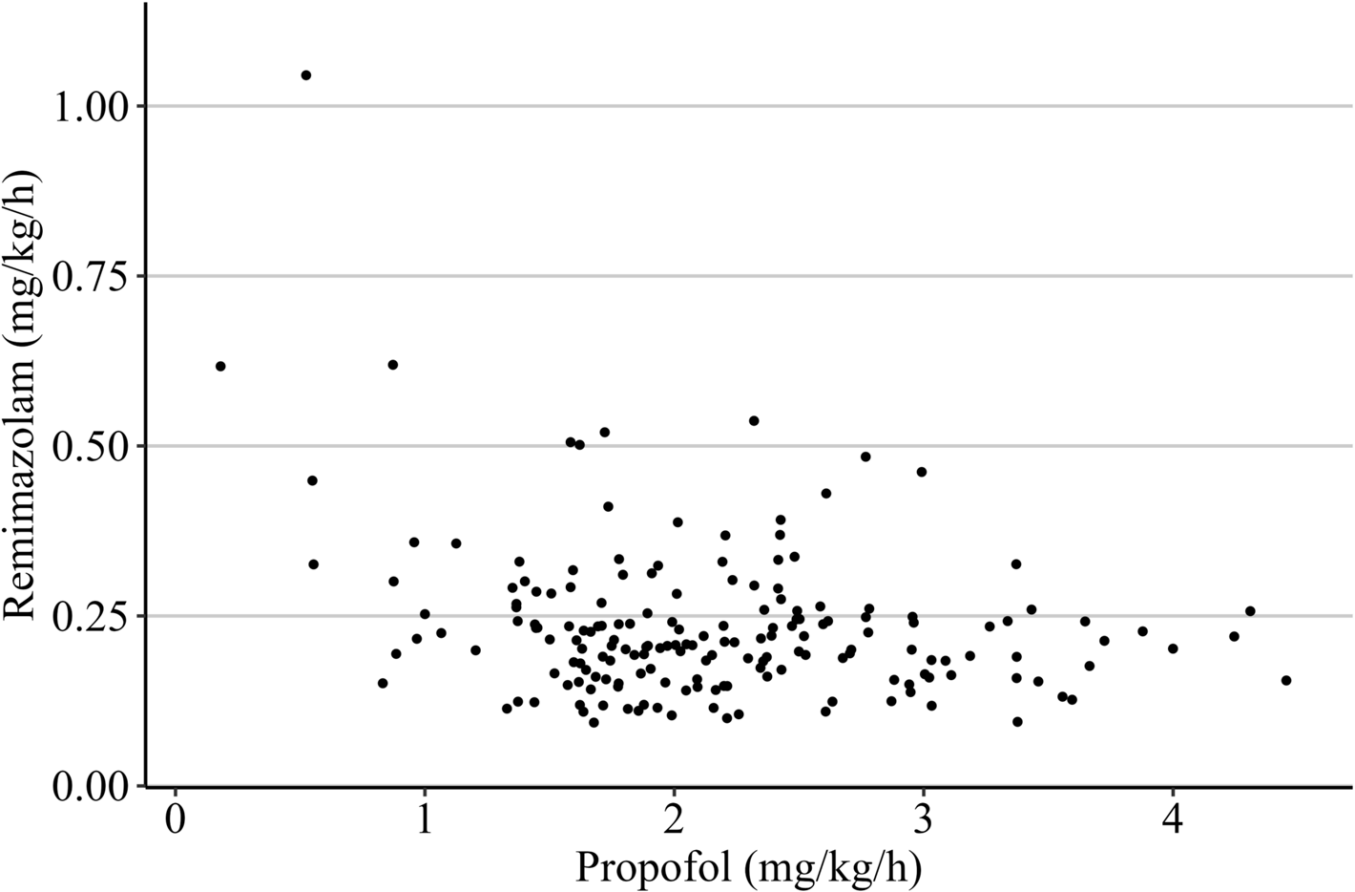
Scatter plot showing the mean intraoperative infusion rates of remimazolam and propofol in the RB + PROP group. The vertical and horizontal axes represent the mean infusion rates of remimazolam (mg/kg/h) and propofol (mg/kg/h) during anesthesia maintenance, respectively. Each point corresponds to an individual patient in the RB + PROP group (*n* = 178)

The intraoperative parameters before and after propensity score matching are summarized in Table 2. There were no significant differences between the groups in terms of fluid volume or opioid administration. The mean infusion rates of remimazolam and propofol in the 178 patients in the RB + PROP group are shown in Fig. 2. The median infusion rates were 0.21 mg/kg/h and 2.1 mg/kg/h for remimazolam and propofol, respectively. Propofol was administered using a TCI system in the majority of patients in both the PROP and RB + PROP groups. The amount of ephedrine administered was significantly lower in the RB group than in the RB + PROP group (4 mg, IQR 0–8 vs. 8 mg, IQR 4–13). Phenylephrine doses were 0 mg (IQR 0–0.10), 0.17 mg (IQR 0–0.42), and 0.10 mg (IQR 0–0.42) in the RB, PROP, and RB + PROP groups, respectively. The RB group received significantly less phenylephrine than the PROP and RB + PROP groups. Flumazenil was administered to 97.3% of the patients in the RB group and 92.0% in the RB + PROP group, with no significant difference (*P* = 0.28). The only difference in intraoperative antiemetic use between the groups was ondansetron. For this drug, significant differences were observed between each group, with the proportion of patients receiving ondansetron being highest in the RB + PROP group (80.0%), followed by the PROP (50.7%) and RB (29.3%) groups.

**Table 2:** Intraoperative variables before and after propensity score matching

	Total cohort				Propensity score-matched cohort			
	Group RB	Group PROP	Group RB + PROP	P	Group RB	Group PROP	Group RB + PROP	P
	*(n = 165)*	(*n* = 403)	(*n* = 178)		(*n* = 75)	(*n* = 75)	(*n* = 75)	
Intraoperative variables								
Anesthesia time (min)	238 (176–320)	262 (209–340)	261 (201–338)	0.024	232 (193–303)	258 (205–321)	263 (206–325)	0.33
Intravenous fluid volume (mL)	1500 (1100–2100)	1550 (1200–2150)	1525 (1100–2100)	0.17	1500 (1100–2075)	1550 (1155–2100)	1500 (1150–1950)	0.77
Urine output (mL)	152 (80–280)	241 (110–600)	200 (99–469)	< 0.001	178 (100–300)	160 (98–448)	255 (105–560)	0.23
Remimazolam (mg/kg/h)	0.72 (0.54–0.94)		0.21 (0.16–0.26)	< 0.001	0.76 (0.58–0.97)		0.21 (0.18–0.25)	< 0.001
Propofol (mg/kg/h)		4.1 (3.6–4.6)	2.1 (1.7–2.6)	< 0.001		3.9 (3.5–4.4)	2.1 (1.7–2.4)	< 0.001
Propofol administration by TCI		397 (98.5%)	168 (94.4%)	< 0.001		75 (100%)	69 (92.0%)	< 0.001
Remifentanil (μg/kg/min)	0.18 (0.13–0.22)	0.19 (0.16–0.23)	0.18 (0.14–0.23)	0.007	0.20 (0.15–0.23)	0.19 (0.16–0.22)	0.17 (0.14–0.22)	0.21
Fentanyl (mg)	0.20 (0.10–0.33)	0.25 (0.15–0.40)	0.30 (0.20–0.40)	0.011	0.20 (0.14–0.35)	0.30 (0.10–0.40)	0.25 (0.12–0.35)	0.7
Use of flumazenil	152 (92.1%)	0 (0%)	163 (91.6%)	< 0.001	73 (97.3%)	0 (0%)	69 (92.0%)	< 0.001
Ephedrine (mg)	4 (0–12)	8 (0.5–12)	8 (0–14)	0.006	4 (0–8)	4 (4–11)	8 (4–13)	0.006
Phenylephrine (mg)	0.05 (0–0.25)	0.05 (0–0.30)	0.08 (0–0.30)	0.57	0 (0–0.10)	0.17 (0–0.42)	0.10 (0–0.42)	0.001
Dopamine (mg)	0 (0–0)	0 (0–0)	0 (0–0)	0.11	0 (0–0)	0 (0–0)	0 (0–0)	0.56
Norepinephrine (mg)	0 (0–0)	0 (0–0)	0 (0–0)	0.003	0 (0–0)	0 (0–0)	0 (0–0)	0.084
Use of ondansetron	45 (27.3%)	191 (47.4%)	138 (77.5%)	< 0.001	22 (29.3%)	38 (50.7%)	60 (80.0%)	< 0.001
Use of droperidol	17 (10.3%)	32 (7.9%)	24 (13.5%)	0.114	8 (10.7%)	7 (9.3%)	11 (14.7%)	0.653
Use of metoclopramide	12 (7.3%)	23 (5.7%)	3 (1.7%)	0.029	8 (10.7%)	4 (5.3%)	1 (1.3%)	0.053
Use of dexamethasone	13 (7.9%)	54 (13.4%)	34 (19.1%)	0.01	9 (12.0%)	8 (10.7%)	16 (21.3%)	0.167

Data are expressed as median (interquartile range), or number of patients (%)

*TCI* Target-controlled infusion

### Clinical outcomes

**Fig. 3:**
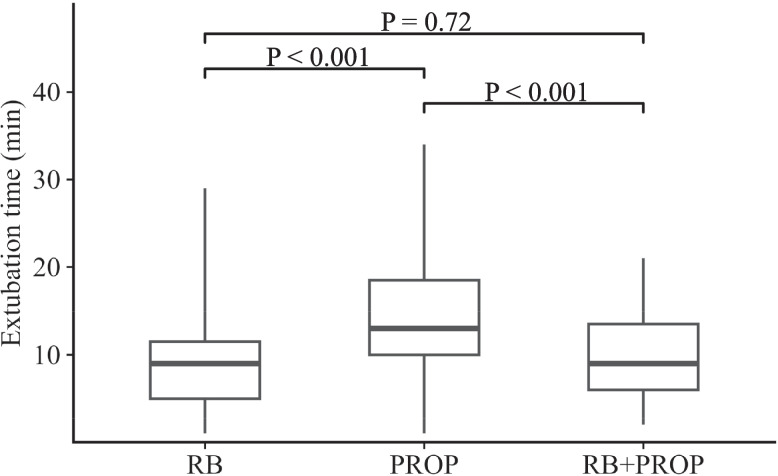
Comparison of extubation times among the three groups. Extubation times are presented as box-and-whisker plots for the RB (remimazolam alone), PROP (propofol alone), and RB + PROP groups (a combination of remimazolam and propofol). Pairwise differences among groups were analyzed using the Steel–Dwass test

The primary outcome, time to extubation, was significantly different among the three groups (Fig. 3). Time to extubation was significantly shorter in the RB group (9.0 min, IQR 5.0–11.5) and the RB + PROP group (9.0 min, IQR 6.0–13.5) than the PROP group (13.0 min, IQR 10.0–18.5) (each *P* < 0.001).

For the secondary outcome, TWA-AUT was highest in the PROP group (2.03 mmHg, IQR 0.53–4.34), followed by the RB + PROP group (1.06 mmHg, IQR 0.28–3.35), and the RB group (0.74 mmHg, IQR 0.14–2.54). A significant difference was observed between the PROP and RB groups (*P* = 0.03) (Table 3).

**Table 3:** Clinical outcomes before and after propensity score matching

	Total cohort				Propensity score-matched cohort			
	Group RB	Group PROP	Group RB + PROP	P	Group RB	Group PROP	Group RB + PROP	P
	(*n* = 165)	(*n* = 403)	(*n* = 178)		(*n* = 75)	(*n* = 75)	(*n* = 75)	
Extubation time (min)	9.0 (6.0–12.0)	13.0 (9.0–18.0)	8.0 (6.0–12.0)	< 0.001	9.0 (5.0–11.5)	13.0 (10.0–18.5)	9.0 (6.0–13.5)	< 0.001
TWA-AUT (mmHg)	0.93 (0.18–3.15)	1.48 (0.30–3.57)	0.99 (0.28–2.76)	0.06	0.74 (0.14–2.54)	2.03 (0.53–4.34)	1.06 (0.28–3.35)	0.036
PONV	28 (17.2%)	57 (14.1%)	24 (13.6%)	0.58	14 (18.7%)	13 (17.3%)	7 (9.3%)	0.23

Data are expressed as median (interquartile range), or number of patients (%)

*TWA-AUT* Time-weighted average of area under the threshold, *PONV* Postoperative nausea and vomiting

The incidence of PONV was 18.7%, 17.3%, and 9.3% in the RB, PROP, and RB + PROP groups, respectively, with no statistically significant difference (*P* = 0.23).

## Discussion

In this retrospective observational cohort study, we compared the extubation times among patients who received maintenance anesthesia with propofol alone, remimazolam alone, or a combination of both. In patients undergoing general anesthesia for non-cardiac surgery, co-administration of propofol and remimazolam resulted in faster awakening than propofol alone and achieved extubation times comparable to those of remimazolam alone. Regarding intraoperative hypotension, a significant difference was observed between the remimazolam and propofol groups. No significant differences in the incidence of postoperative PONV were observed among the three groups.

A study of patients undergoing meningioma resection reported that a prolonged extubation time of ≥ 35 min was associated with an increased risk of postoperative pneumonia [2]. Moreover, prolonged extubation has been associated with longer operating room stays [21], suggesting that early extubation plays a crucial role in reducing postoperative complications and healthcare costs. Previous reports have shown that the co-administration of remimazolam and propofol during gastroscopy allows for a reduced dose of propofol while achieving adequate sedation and shortening the time to awakening [12]. Furthermore, under general anesthesia without flumazenil antagonism, the median time to awakening has been reported as 13 min [22]. In the present study, flumazenil was administered in nearly all cases involving remimazolam; therefore, its effects could not be evaluated directly. Nonetheless, flumazenil administration may have contributed to the earlier awakening observed compared with propofol alone. No cases of suspected re-sedation were observed following flumazenil administration. However, some studies have reported that using flumazenil after high-dose remimazolam administration may increase the risk of re-sedation [15]. Whether co-administration with propofol, which may reduce the required dose of remimazolam, can mitigate this risk remains unclear, and further research is warranted. In the RB + PROP group, propofol was continuously infused in combination with remimazolam during surgery; however, propofol infusion was discontinued earlier than remimazolam, with a median difference of 4.0 min (*P* < 0.001) (Supplementary Fig. 1). This difference likely reflects the anesthesiologists’ clinical decision to taper and discontinue propofol slightly earlier to allow its effect to diminish towards the end of surgery, thereby facilitating smooth emergence. The timing of neuromuscular blockade reversal may also have affected extubation time. After surgery, 38, 25, and 17 of the 75 patients in the RB, PROP, and RB + PROP groups, respectively, were administered a neuromuscular blockade reversal agent. The median times from the end of anesthetic administration to the introduction of a neuromuscular blockade reversal agent were 2.5 (IQR 1.0–5.0), 2.0 (IQR 1.0–4.0), and 2.0 (IQR 0–4.0) minutes in the RB, PROP, and RB + PROP groups, respectively. No significant differences were found among the groups in the time from the end of anesthetic administration to the introduction of the reversal agent (*P* = 0.83) (Supplementary Fig. 2), suggesting that the impact of this factor on the study outcomes was minimal.

General anesthesia with remimazolam is associated with a lower incidence of hypotension than with propofol [23]. In the present study, despite the lower dose of phenylephrine administered to the RB group, the severity of hypotension was reduced compared to that in the PROP group.

When remimazolam is combined with propofol for general anesthesia, the incidence of hypotension increases with increasing doses of remimazolam [24]. In contrast, during hysteroscopic procedures, the co-administration of remimazolam with propofol is associated with a lower incidence of hypotension than propofol alone [25]. In this study, no significant difference in the severity of hypotension was observed between the PROP and RB + PROP groups. Overall, the hemodynamic effects of remimazolam combined with propofol may differ based on the surgical context and dosing strategy.

Propofol is an intravenous anesthetic agent with antiemetic properties, and the incidence of postoperative nausea and vomiting is lower than that of inhalational anesthetics [26]. In gynecological surgeries under general anesthesia using remimazolam, the co-administration of low-dose propofol reduces the incidence of PONV [27]. A pior retrospective cohort study reported a higher incidence of PONV with remimazolam than with propofol [18]. As remimazolam is rapidly metabolized, its intrinsic antiemetic effect may be attenuated by antagonism with flumazenil [28]. In contrast, remimazolam is associated with a lower incidence of PONV than inhalational anesthetics [29]. Moreover, a meta-analysis found no significant difference in the incidence of PONV between remimazolam and propofol [14]. In the present study, no significant difference in the incidence of PONV was observed between groups.

These findings suggest that intravenous anesthetics, regardless of the specific agent used, tend to result in a lower incidence of PONV than inhalational anesthesia, and the differences between agents may be minimal. In the present study, the rate of ondansetron use differed significantly between groups, which may reflect the influence of potential confounding factors that were not fully controlled. Future comparative studies should further consider dosage strategies, perioperative antiemetic management, and patient risk stratification.

This study had several limitations. First, because this was a single-center study, the generalizability of the results may be limited. Second, EEG monitoring was performed at the discretion of the attending anesthesiologist. EEG monitoring with either Bispectral Index™ or SedLine® was conducted in almost all cases, and there were no significant intergroup differences in Bispectral Index and Patient State Index values at the discontinuation of the anesthetic maintenance drug used during surgery (Supplementary Table 1). Furthermore, we found no significant between-group differences in the percentage of intraoperative time during which BIS or PSI values remained within the appropriate range. The proportion of time with PSI values > 50 was longer in the RB group than in the PROP group (*P* = 0.01); however, there was no significant difference between the PROP and RB + PROP groups (*P* = 0.05). It should be noted that the effects of remimazolam monotherapy and combination therapy with propofol on EEG patterns remain unclear, and it is possible that the depth of anesthesia differed between the groups. Third, the timing of neuromuscular blockade reversal varied among attending anesthesiologists. Residual neuromuscular blockade could potentially place patients at increased risk [30]. Therefore, it is critically important that the effects of neuromuscular blocking agents are fully reversed, either spontaneously or with reversal agents, before emergence from anesthesia. Finally, no standardized protocol was established for the administration of remimazolam and propofol in patients under general anesthesia. The dosing ratios of these agents varied among anesthesiologists. To prevent intraoperative awareness and ensure patient safety, careful patient observation and individualized management are essential. Further studies are required to determine the optimal dosing strategy for this drug combination.

## Conclusions

The combination of remimazolam and propofol, in conjunction with antagonism by flumazenil, may promote earlier recovery than propofol alone, while maintaining hemodynamic stability during anesthesia maintenance. This administration strategy represents a novel option for safe and efficient anesthesia management that avoids delayed emergence; however, its clinical utility remains to be validated in large-scale prospective studies.

## Supplementary Information

Supplementary Material 1: Supplementary Fig. 1. Box-and-whisker plots showing the distribution of time intervals from discontinuation of remimazolam and propofol to extubation in the RB + PROP group (*n* = 75). Each pair of corresponding cases is connected by a line to illustrate the within-patient differences. The Wilcoxon signed-rank test was applied to compare the paired data.

Supplementary Material 2: Supplementary Fig. 2. Box-and-whisker plots showing the time from the end of anesthetic administration to the initiation of a neuromuscular blockade reversal agent in the RB, PROP, and RB + PROP groups

Supplementary Material 3: Supplementary Table 1. Summary of Intraoperative EEG Monitoring Data. Data are expressed as median (interquartile range), or number of patients (%). EEG, electroencephalogram; BIS, bispectral index; PSI, patient state index

## Data Availability

The datasets used and/or analyzed in the current study are available from the corresponding author upon reasonable request.

## Abbreviations

TCI: Target-Controlled Infusion
EEG: Electroencephalogram
BIS: Bispectral Index
PSI: Patient State Index
BMI: Body Mass Index
ASA-PS: American Society of Anesthesiologists Physical Status
PONV: Postoperative Nausea and Vomiting
MAP: Mean Arterial Pressure
TWA-AUT: Time-Weighted Average Area Under the Threshold
