# Supplementary figures and images for: Association between a remimazolam–propofol combination for maintenance of anesthesia and extubation time: a propensity score analysis
Source: JA Clin Rep. 2025 Dec 4;12:5. doi: 10.1186/s40981-025-00836-2 (PMC12779813; doi:10.1186/s40981-025-00836-2)

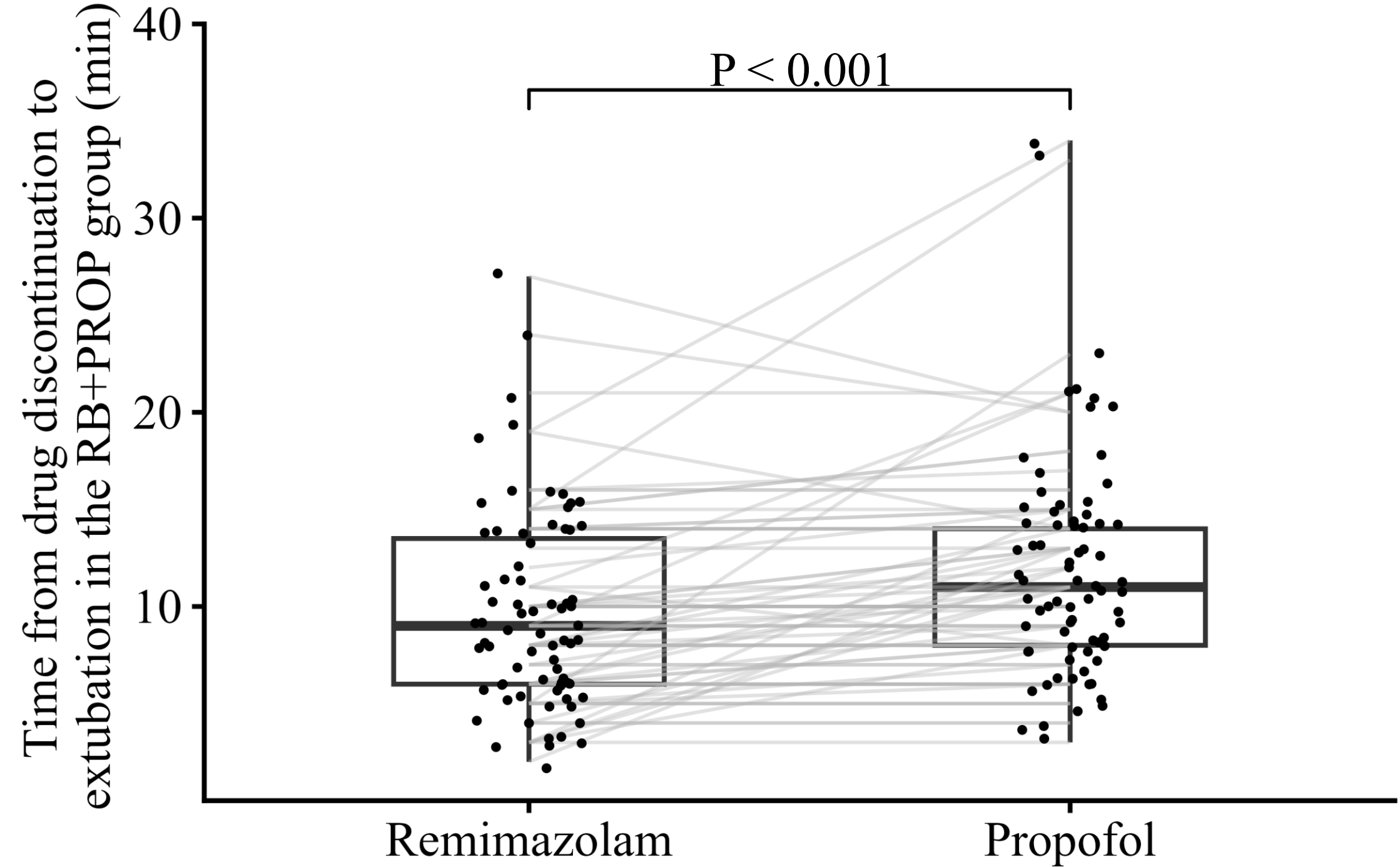

Supplement: Supplementary file 1 — Supplementary Material 1: Supplementary Fig. 1. Box-and-whisker plots showing the distribution of time intervals from discontinuation of remimazolam and propofol to extubation in the RB + PROP group (n = 75). Each pair of corresponding cases is connected by a line to illustrate the within-patient differences. The Wilcoxon signed-rank test was applied to compare the paired data. [file 40981_2025_836_MOESM1_ESM.pdf]

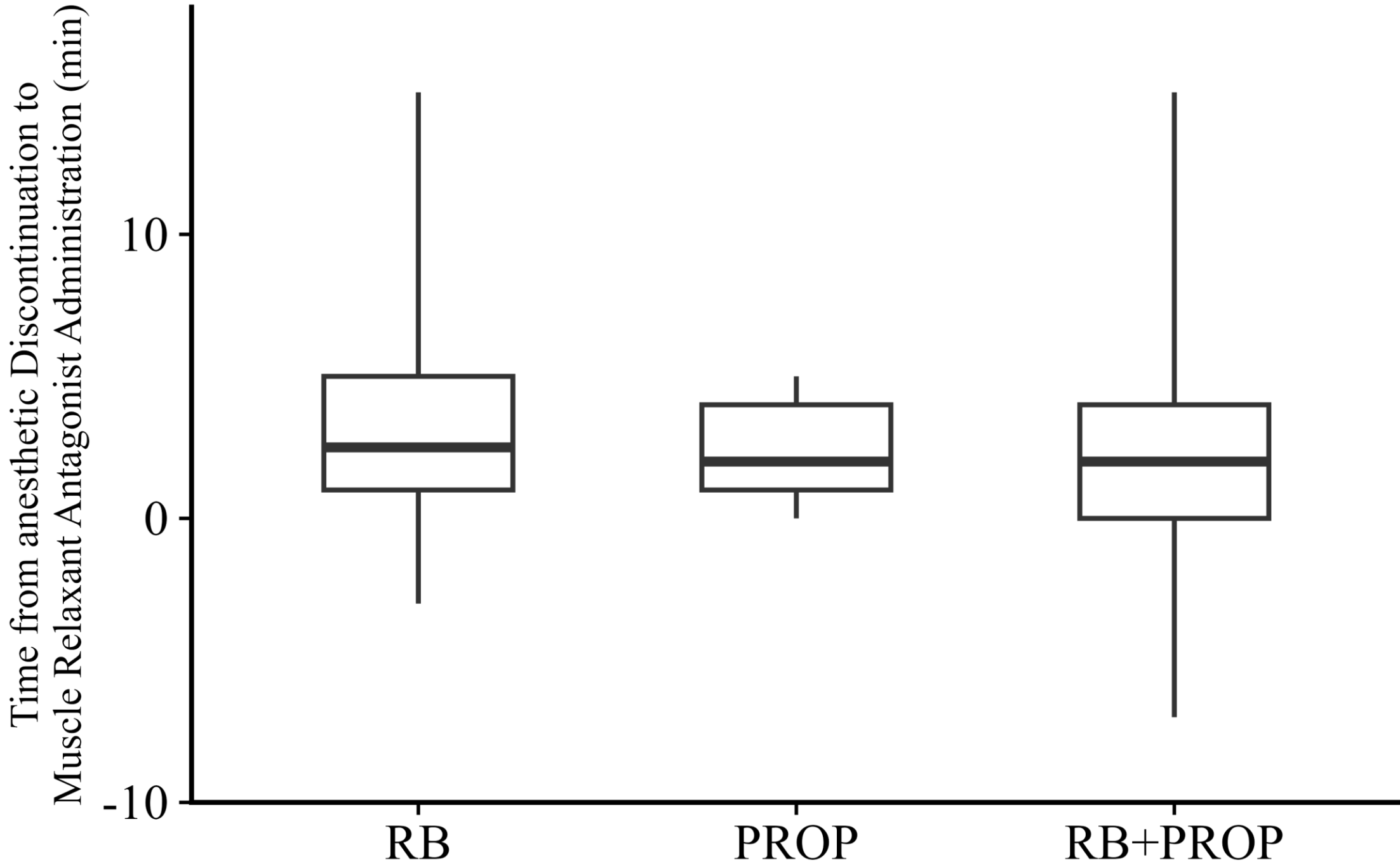

Supplement: Supplementary file 2 — Supplementary Material 2: Supplementary Fig. 2. Box-and-whisker plots showing the time from the end of anesthetic administration to the initiation of a neuromuscular blockade reversal agent in the RB, PROP, and RB + PROP groups [file 40981_2025_836_MOESM2_ESM.pdf]
